# Differential expression of CD44 and CD24 markers discriminates the epitheliod from the fibroblastoid subset in a sarcomatoid renal carcinoma cell line: evidence suggesting the existence of cancer stem cells in both subsets as studied with sorted cells

**DOI:** 10.18632/oncotarget.14777

**Published:** 2017-01-21

**Authors:** Chin-Hsuan Hsieh, Shih-Chieh Hsiung, Chi-Tai Yeh, Chih-Feng Yen, Yah-Huei Wu Chou, Wei-Yi Lei, See-Tong Pang, Cheng-Keng Chuang, Shuen-Kuei Liao

**Affiliations:** ^1^ Division of Uro-oncology, Department of Surgery, Chang Gung Memorial Hospital, Taoyuan, Taiwan; ^2^ Department of Medical Research and Development, Chang Gung Memorial Hospital, Taoyuan, Taiwan; ^3^ Department of Obstetrics and Gynecology, Chang Gung Memorial Hospital, Taoyuan, Taiwan; ^4^ Cancer Center, Taipei Medical University-Shuang Ho Hospital, Taipei, Taiwan; ^5^ School of Medicine, Chang Gung University, Taoyuan, Taiwan; ^6^ Graduate Institute of Clinical Medical Sciences, Chang Gung University, Taoyuan, Taiwan; ^7^ Department of Medical Science and College of Medicine, Tzu-Chi University, and Department of Internal Medicine, Tzu-Chji General Hospital, Hua-lien, Taiwan; ^8^ The Ph.D. Program for Cancer Biology and Drug Discovery, Taipei Medical University, Taipei, Taiwan; ^9^ Vectorite Biomedica Inc., Taipei, Taiwan

**Keywords:** cancer stem cells, sarcomatoid renal cell carcinoma, epithelioid and fibroblastoid subsets, CD24, CD44

## Abstract

Epithelioid and fibroblastoid subsets coexist in the human sarcomatoid renal cell carcinoma (sRCC) cell line, RCC52, according to previous clonal studies. Herein, using monoclonal antibodies to CD44 and CD24 markers, we identified and isolated these two populations, and showed that CD44^bright^/CD24^dim^ and CD44^bright^/CD24^bright^ phenotypes correspond to epithelioid and fibroblastoid subsets, respectively. Both sorted subsets displayed different levels of tumorigenicity in xenotransplantation, indicating that each harbored its own cancer stem cells (CSCs). The CD44^bright^/CD24^bright^ subset, associated with higher expression of *MMP-7, -8* and *TIMP-1* transcripts, showed greater migratory/invasive potential than the CD44^bright^/CD24^dim^ subset, which was associated with higher expression of *MMP-2, -9* and *TIMP-2* transcripts. Both subsets differentially expressed stemness gene products c-Myc, Oct4A, Notch1, Notch2 and Notch3, and the RCC stem cell marker, CD105 in 4-5% of RCC52 cells. These results suggest the presence of CSCs in both sRCC subsets for the first time and should therefore be considered potential therapeutic targets for this aggressive malignancy.

## INTRODUCTION

Renal cell carcinoma (RCC) accounts for approximately 3% of human malignancies, has a high metastatic index at diagnosis and a high rate of relapse [1, 2]. Histological types include clear cell, papillary, chromophobe, collecting duct, and unclassified RCCs. Sarcomatoid RCC (sRCC) is a subtype that can transform from any histological type, except unclassified RCCs [3]. Compared to other subtypes, sRCCs are associated with poorer prognosis, higher metastatic and local recurrence rates, shorter survival intervals, and relative resistance to multiple forms of systemic therapy [4, 5]. However, sRCCs constitute only 1 to 5% of all RCC cases [6, 7]. While some cellular and molecular characteristics of sRCC have gradually emerged [8, 9], our understanding of its conversion (from epithelioid to fibroblastoid cells, to clear cells, and/or vice versa), genetics and cellular heterogeneity in tumor progression is still far from complete. A fuller understanding of the molecular mechanisms and pathobiology of sRCC will help provide a scientific basis for developing novel approaches to treatment.

The concept of cancer stem cells (CSCs) has been proposed to help explain tumorigenesis and tumor progression. This hypothesis emphasizes that the stem cell hierarchy is constructed by heterogeneous tumor cells, within which exists only a rare specific subpopulation of cancer cells with stem cell properties [10, 11]. This subpopulation, referred to as CSCs or tumor-initiating cells (TICs), has the ability to self-renew and give rise to differentiated progenies of cancer cells that contribute to tumor initiation and reestablish tumor heterogeneity [10]. Several studies indicate that CSCs might cause resistance to chemotherapy and radiotherapy [12] and cell-based immunotherapeutic effectors [13]. Putative CSCs have been identified in several human tumors based on the expression of a specific molecule or combination of molecules (e.g., CD133, CD44, CD166, aldehyde dehydrogenases), including the first identified acute myeloid leukemia [14], and a few solid tumors such as brain [15], breast [16], gastrointestinal [13] and prostate cancer [17]. CSCs have been identified in some solid tumors to be located in the CD44^bright^/CD24^dim/−^ subset [13, 15, 16].

Only a few studies have indicated possible CSC marker(s) in RCC [18–21]. Addla *et al*. used the Hoechst 33342 dye efflux assay to isolate epithelial side population (SP) and non-SP cells from normal and malignant human renal tissues and found that only renal SP cells had a high proliferative capacity and stem-like properties [18]. To our knowledge, there are no published reports examining the dual expression of two combinations of CSC markers, CD44 and CD24, in sRCC. CD44 is considered a potential CSC marker in breast, prostate, pancreas, ovarian, and colorectal cancers [16, 17, 22–24]. CD24 is emerging as a marker of malignant cells and an effector of hypoxia-inducible factor (HIF)-1-driven tumor growth and metastasis [25], although the association of CD24 and CSCs is not uniform [26, 27]. The combination of CD44 and CD24 has been investigated in breast carcinoma [16] and pancreatic cancer [24].

The present study sought to use these two CD markers to isolate two subsets of the sRCC cell line, RCC52, which has previously been established and characterized in our laboratory [28, 29]. In this study, most results obtained from the cytofluorometrically sorted cells were similar to those obtained with the clonal sublines morphologically and phenotypically, but not to those of xenotransplantation, *in vitro* colony forming studies, or *in vitro* migratory/invasive assays [29]. We found that the CD44^bright^/CD24^bright^ sorted subset invariably resulted in much larger xenografts as a function of time, when compared with those developed by either the parental cells or the CD44^bright^/CD24^dim^ sorted subset. These results indicate that both sorted cell subsets of the RCC52 cell line/tumor harbored different classes of CSCs. The results obtained with sorted cells are likely to represent the true picture of this cell line/tumor in *in vivo* situations, since the spectra of whole cell populations of each sorted subset were all being considered and analyzed. This is in contrast to our previous findings in which only small numbers of clonal isolates were investigated; these clonal sublines from the two morphologically distinct subsets were likely under representation of RCC52 cells [29].

## RESULTS

### Differential expression of surface CD44 and CD24 by a panel of RCC cell lines

**Figure 1:**
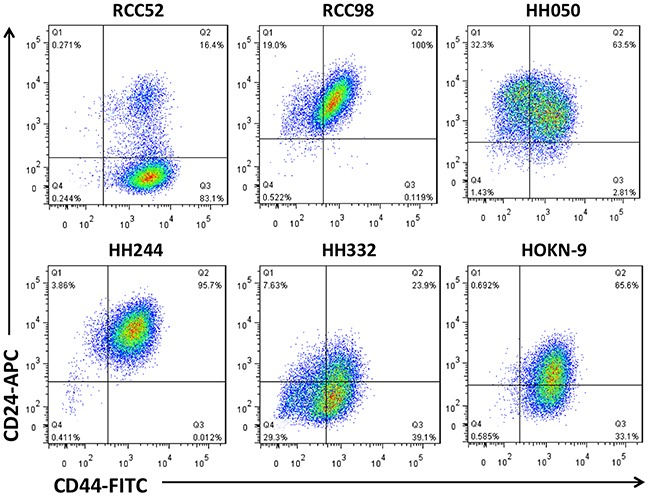
Two color cytofluorometric analysis on six different histologic RCC cell lines Six different histologic RCC cell lines, sarcomatoid RCC52, chromophobe RCC98, papillary HH050, tubular HH244, clear cell HH332 and HOKN-9, were tested with anti-CD44 and anti-CD24 mAbs conjugated with FITC and APC respectively, as test reagents.

Results of two color-analyses in cytofluorometry revealed that only the sRCC cell line RCC52 showed two discrete masses of cells with the phenotypes of CD44^bright^/CD24^bright^ and CD44^bright^/CD24^dim^, respectively (Figure 1). Conversely, only one mass of cell population with the phenotype of CD44^bright^/CD24^bright^ (RCC98, HH050 and HH244), or similar but with much less proportion of CD44^bright^/CD24^bright^ (HH322 and HOKN-9) cells, was detected in the 5 remaining RCC cell lines, including 2 clear cell, 1 chromophobe, 1 papillary and 1 tubular.

### Differential expression of surface CD44 and CD24 by the RCC52 epithelioid and fibroblastoid sublines

**Figure 2:**
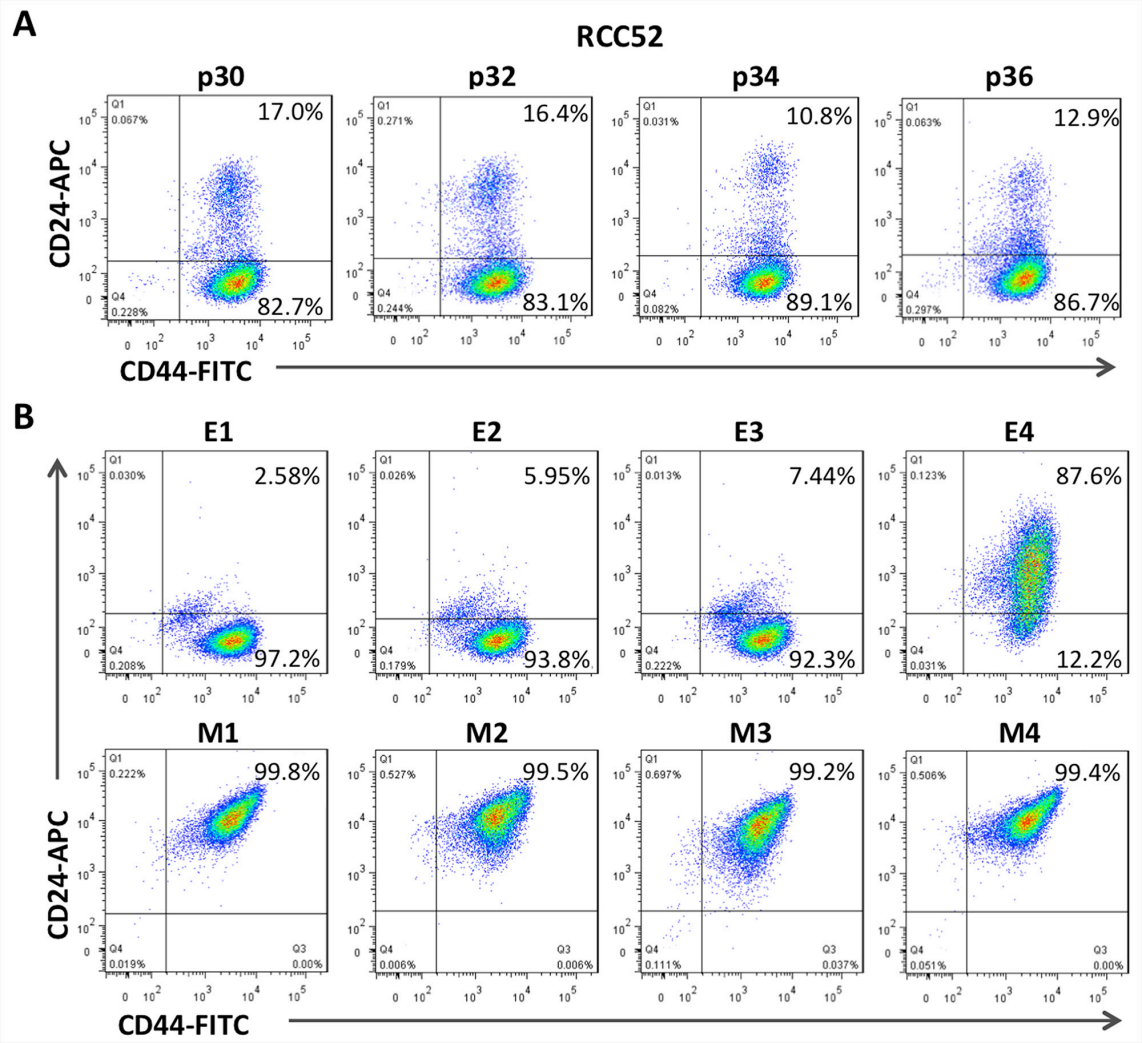
Two color cytofluorometric analysis on RCC52 cells and the clonal sublines Immunophenotypic profiles of the parental, epithelioid (E1~E4) and fibroblastoid (M1~M4) clonal sublines of the RCC52 cell line as determined by two color analysis with anti-CD44 and anti-CD24 mAbs conjugated with FITC and APC respectively. **A**. The expression of CD44/CD24 profile of different passage of RCC52 cells. **B**. The expression of CD44/CD24 profile in epithelioid and fibroblastoid sublines.

As shown in Figure 1, only RCC52 cells showed two discrete masses of cell populations. The ratio of the cell number in these two morphologically distinct subsets in RCC52 cells was consistently 6:1 in four different *in vitro* passages (85.4 ± 3.0% of the CD44^bright^/CD24^dim^ cells *vs*. 14.3 ± 2.9% of the CD44^bright^/CD24^bright^ cells; Figure 2A).

When the four fibroblastoid clonal sublines previously isolated and established *in vitro* [29] were tested, we consistently obtained one mass on the plot with a phenotype of CD44^bright^/CD24^bright^ at the upper right (Figure 2B). However, analysis of the 3/4 epithelioid sublines revealed a major mass at the lower right position, consistent with the phenotype of CD44^bright^/CD24^dim^ found in one of the two masses in the parental RCC52 cells. The remaining E4 epithelioid subline exhibited an elongated mass containing both CD44^bright^/CD24^bright^ and CD44^bright^/CD24^dim^ masses in fusion (Figure 2B). Repeated experiments conducted with E4 and other sublines at the same or different passage levels showed similar results.

### *In vitro* growth and immunophenotypic features of the two sorted RCC52 subsets

**Figure 3:**
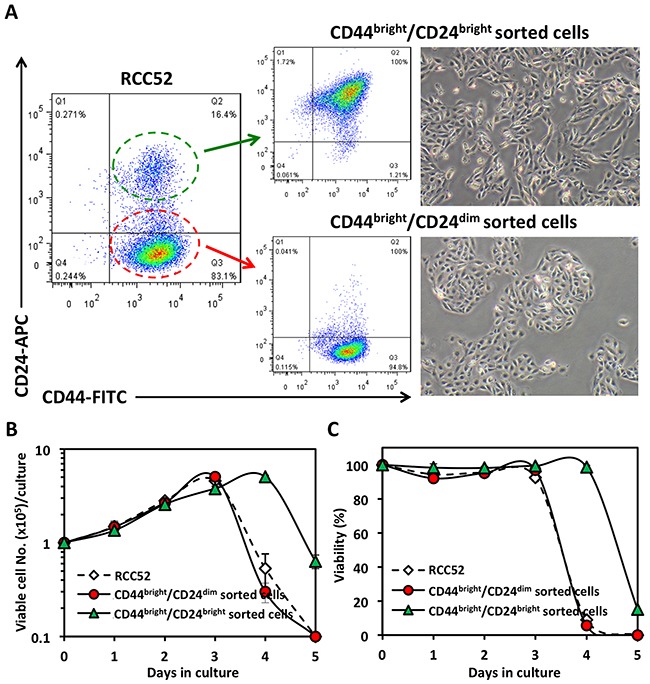
Morphology and *in vitro* growth patterns of sorted RCC52 subsets **A**. Cells were sorted using mAbs to CD44 and CD24. Live monolayer cultures of CD44^bright^/CD24^dim^ sorted cells and CD44^bright^/CD24^bright^ sorted cells are shown in the right panel. Original magnification, × 100. **B, C**. Growth curves of the RCC52 parental and CD44/CD24 sorted cells cultured *in vitro* in which the total viable cell number/dish and % cell viability at each time point are indicated. Each time point represents mean ± SD of triplicate wells.

We next used mAbs against CD44 and CD24 to sort these two cell populations, and named them CD44^bright^/CD24^dim^ sorted and CD44^bright^/CD24^bright^ sorted cells, respectively, from the parental cells (Figure 3A). The two sorted cell populations exhibited dissimilar cell morphologies and growth patterns, in that the CD44^bright^/CD24^dim^ sorted cells showed epithelioid cell morphology, while the CD44^bright^/CD24^bright^ sorted cells showed fibroblastoid cell morphology, as expected (Figure 3A). In the *in vitro* growth assay, the CD44^bright^/CD24^dim^ sorted cells exhibited a similar growth pattern to the parental RCC52 cells (Figure 3B and 3C). Of note, cell viability was maintained up to 90% during the exponential phase for both the parental and the CD44^bright^/CD24^dim^ sorted cells, and once confluence was reached at about day 3, monolayer cells started to detach from the bottom of culture vessels. However, in the CD44^bright^/CD24^bright^ sorted cells, the confluence or saturation state was sustained one day longer than the CD44^bright^/CD24^dim^ sorted cells before they became rapidly detached from the vessel surface.

Immunophenotypic differences between the CD44^bright^/CD24^dim^ sorted and the CD44^bright^/CD24^bright^ sorted cells were further compared by cytofluorometric analysis (Table 1). In line with our previous observations [28, 29], HLA class I was not detected on the cell surface or in the cytoplasm of either CD44/CD24 sorted cells. Moreover, surface E-cadherin was detected in the CD44^bright^/CD24^dim^ sorted but not in the CD44^bright^/CD24^bright^ sorted cells.

**Table 1:** Surface and cytoplasmic expression of selected antigens on the RCC52 parental and CD44/CD24 sorted cells as determined by cytofluorometric analysis

Antigen	% Positive cells (MFI)		
	RCC52	CD44^bright^/CD24^dim^ sorted cells	CD44^bright^/CD24^bright^ sorted cells
***Surface***			
HLA class I	-	-	-
E-cadherin	17.2 (175)	18.4 (171)	-
N-cadherin	-	-	-
EpCAM	-	-	-
Le^y^	-	-	-
BH8.23	-	-	-
NMIg/Isotype control	-	-	-
***Cytoplasmic***			
HLA class I	-	-	-
Hsp70	-	-	-
EMA	-	-	-
E-cadherin	-	-	-
N-cadherin	40.5 (283)	46.1 (297)	49.5 (383)
BH8.23	96.1 (1105)	96.8 (1221)	96.1 (1017)
S100-A4	41.7 (140)	35.3 (154)	24.4 (148)
Vimentin	97.3 (3032)	92.9 (3764)	96.7 (4765)
NMIg/Isotype control	-	-	-

Abbreviations: MFI, mean fluorescence intensity in arbitrary units; -, signifies a negative result, with % positive <10%; HLA class I, human leukocyte antigen class I; EpCAM, epithelial cell adhesion molecule; NMIg, normal mouse immunoglobulin; HSP70, heat shock proteins 70; EMA, epithelial membrane antigen; Le^y^, Lewis Y antigen.

### Migration and invasion ability of the two sorted RCC52 subsets

**Figure 4:**
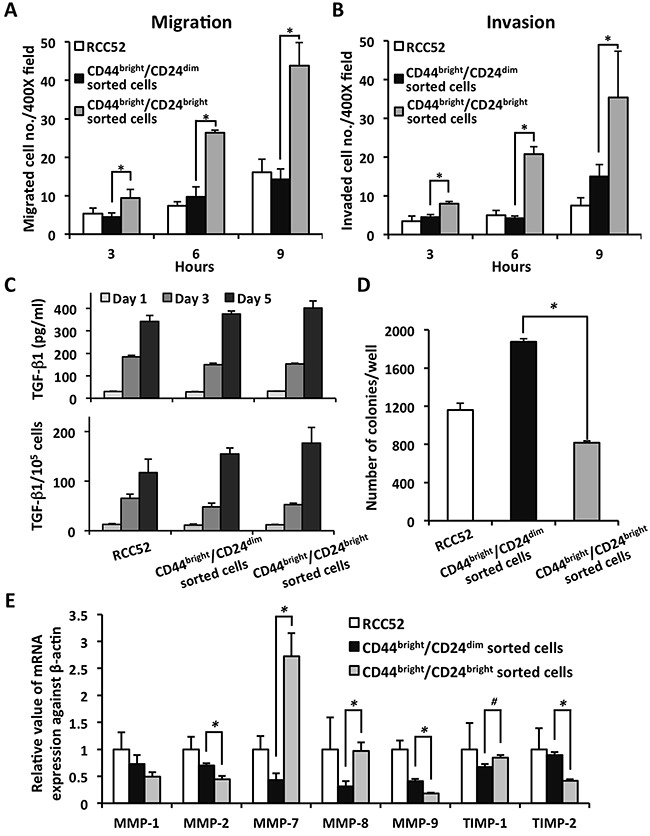
*In vitro* migration/invasion potential, TGF-β1 secretion, colony-forming-ability and *MMPs* expression of the sorted RCC52 subsets The migration **A**. and invasion **B**. abilities of parental and sorted RCC52 cells were conducted in 3 time points, 3, 6 and 9 h. Cells in three randomly selected microscopic fields (× 400) were counted. Each time point was done in triplicate. *p < 0.01. **C**. For the TGF-β1 secretion levels, the spent media were collected from the cell supernatants at day 1, 3 and 5, and assayed for TGF-β1 levels by ELISA. Results were expressed as mean ± SD. **D**. Anchorage-independent clonogenicity was determined by culturing cells in 0.5% low-melting agarose supplied with complete RPMI-1640 medium. 5,000 cells were seeded in a 6-well plate and incubated for 20 days to allow colony formation. Cell colonies were visualized by 5% crystal violet staining. Results were expressed as mean ± SD. *p < 0.01. **E**. qPCR measurement of relative *MMP* mRNA expression in cells as normalized with β-actin. Results were expressed as mean ± SD of triplicate determinations. *p < 0.01; ^#^*p* < 0.05.

Results of *in vitro* migration and invasion assays showed that both the migrated and invaded cell numbers in the CD44^bright^/CD24^bright^ sorted cells were higher than in the parental RCC52 cells and the CD44^bright^/CD24^dim^ sorted cells at each of the three time points tested (3, 6 and 9 h; *p <* 0.01) (Figure 4A and 4B).

### Secretion of TGF-β1 by the two sorted RCC52 subsets

All three groups of tested cells secreted TGF-β1 in the spent medium in a time-dependent manner. The CD44^bright^/CD24^bright^ sorted cells secreted increased amounts of TGF-β1 over a period of five days (401.8 ± 31.2 pg/ml) compared to the CD44^bright^/CD24^dim^ sorted cells (375.9 ± 12.3 pg/ml); the parental cells (341.8 ± 27.0 pg/ml) secreted the least (Figure 4C). There was no significant difference in TGF-β1 secretion between these two sorted subsets (*p* > 0.05), when they were cultured *in vitro* under similar conditions independently.

### Anchorage-independent colony forming ability of the two sorted RCC52 subsets

Results of the soft agar colony forming assay showed that the CD44^bright^/CD24^dim^ sorted cells gave rise to the highest numbers of colonies (1875.5 ± 33.6/well); this was significantly higher than the CD44^bright^/CD24^bright^ sorted cells (817.5 ± 19.4/well; *p* < 0.01). Colony numbers from the parental RCC52 cells fell between the two sorted subsets (1160.7 ± 71.8/well) (Figure 4D).

### Transcript expression of selective *MMPs* and their inhibitors by the two sorted RCC52 subsets

Results of the quantitative real-time PCR (qPCR) assay are shown in Figure 4E. Significantly higher *MMP-2*, *-9* and *TIMP-2* mRNA expressions were detected in the CD44^bright^/CD24^dim^ sorted cells compared to the CD44^bright^/CD24^bright^ sorted cells (*p* < 0.01). The CD44^bright^/CD24^bright^ sorted cells showed significantly higher *MMP-7*, *-8* and *TIMP-1* mRNA expression than the CD44^bright^/CD24^dim^ sorted cells (*p* < 0.05). *MMP-3* and *-13* mRNA expressions were undetectable in RCC52 parental cells and in either sorted subset (data not shown).

### Xenotransplantation of the two sorted RCC52 subsets

**Figure 5:**
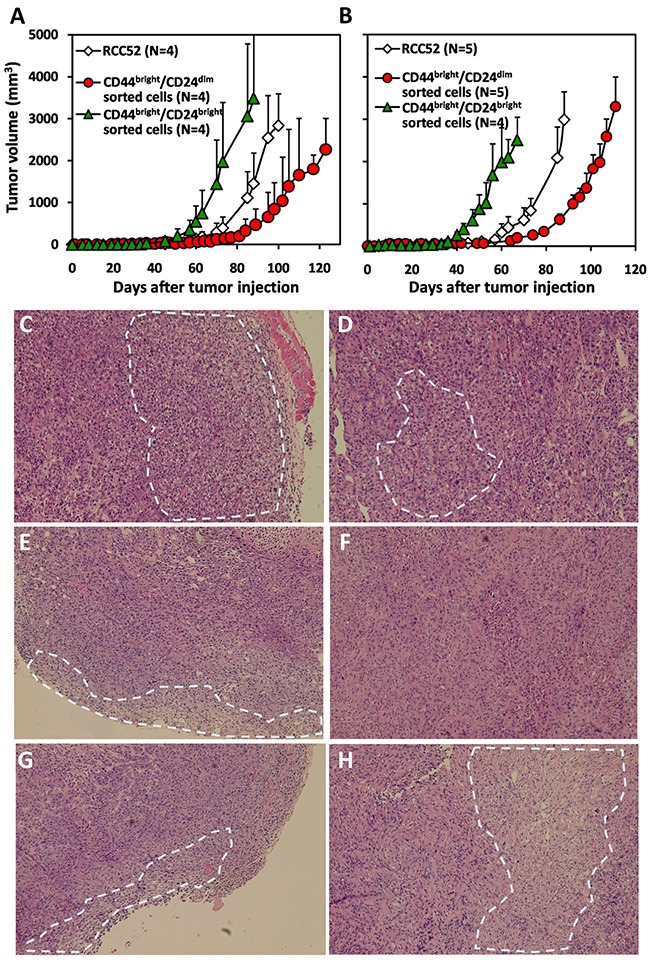
Xenotransplantation of the xenografts of sorted RCC52 subsets Cells (**A**. 1×10^6^/0.1 ml PBS; **B**. 5×10^6^/0.1 ml PBS) of parental RCC52 cells, CD44^bright^/CD24^dim^ sorted cells and CD44^bright^/CD24^bright^ sorted cells were injected subcutaneously in NOD/SCID mice at the hind leg. The animals were examined for the growth of tumors every 2 days for a period of 74 to 132 days. The mean curves with standard deviations are also illustrated at each time point in dotted lines. Note that 4 or 5 animals were used for subcutaneous injection of each experimental animal group. H&E staining profiles on the sections of a representative RCC52 xenograft **C, D**. a CD44^bright^/CD24^bright^ sorted cell xenograft **E, F**. and a CD44^bright^/CD24^dim^ sorted cell xenograft **G, H**. are shown. Figure C, E and G showed the outer region of the tumor mass. Figure D, F and H showed the inner region of the tumor mass. The white dotted circle was the representative clear cell area. Original magnification, × 100.

CD44^bright^/CD24^dim^ sorted and CD44^bright^/CD24^bright^ sorted cells were able to develop tumors at the injection sites with 1×10^6^ and 5×10^6^ cells injected subcutaneously in NOD/SCID mice (Figure 5). In mice injected with 1×10^6^ tumor cells, the tumor volume generated by the CD44^bright^/CD24^dim^ sorted cells was 123.9 ± 28.5 mm^3^ at day 74; tumor volume generated by parental RCC52 cells was 393.4 ± 268.8 mm^3^ (Figure 5A). Tumor volume generated by CD44^bright^/CD24^bright^ sorted cells was much larger (1971.6 ± 1415.8 mm^3^ at day 74). A similar trend of results was obtained with animals after 5×10^6^ cells were injected in NOD/SCID mice, and tumor volumes were proportionally larger with this higher dose of tumor cells (Figure 5B).

### Histochemical and immunostaining patterns of xenografts resulting from injection of cultured cells of the two sorted RCC52 subsets to NOD/SCID mice

Histochemical staining of tumor sections from the xenografts injected with parental RCC52 cells showed that the majority of tumor cells were of sarcomatoid histology, while only focal areas exhibited clear cell morphology (Figure 5C and 5D). The clear cell areas were mostly found in the outer region of the tumor (Figure 5C). This was also the case in tumor sections from xenografts injected with CD44^bright^/CD24^dim^ sorted cells and with CD44^bright^/CD24^bright^ sorted cells, although a slightly higher frequency of clear cell areas were found in the former (Figure 5E~5H).

A subtle difference was noted in the xenografts from the two sorted cell subsets. Those from the CD44^bright^/CD24^dim^ sorted cells showed relatively larger clear cell areas randomly distributed in the tumor periphery with smaller clear cell areas centrally. Those from CD44^bright^/CD24^bright^ sorted cells, however, showed smaller clear cell areas sparsely distributed in the tumor periphery with even smaller clear cell areas scattered centrally. Therefore, CD44^bright^/CD24^dim^ sorted cells had a greater ability to develop clear cell morphology than CD44^bright^/CD24^bright^ sorted cells.

**Figure 6:**
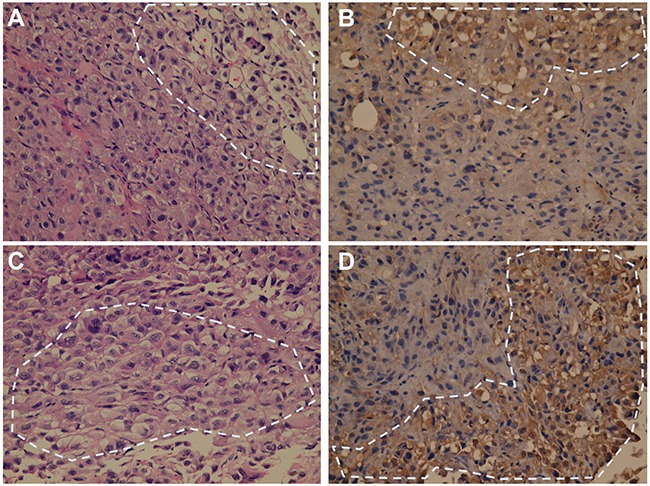
Immunostaining of PAX2 on tumor sections of the representative xenografts resulting from sorted RCC52 subsets H&E staining profiles **A, C**. and immunostaining profiles for PAX2 positivity **B, D**. on the sections of a xenograft resulting from injection with CD44^bright^/CD24^bright^ sorted cells (A, B) and a xenograft from injection with CD44^bright^/CD24^dim^ sorted cells (C, D) were shown. Note that positive staining for PAX2 was detected in brown color only in the clear cell areas among the sea of sarcomatoid components. Representative clear cell area is demarcated with the dotted circle. Original magnification, × 400.

We also used PAX2 marker to verify the clear cell components in NOD/SCID mouse xenografts. Immunostaining xenografts with anti-PAX2 showed positive reactivity with the clear cell component, but negative with the sarcomatoid component (Figure 6). Clear cell areas staining positively for PAX2 were occasionally found in xenografts from CD44^bright^/CD24^bright^ sorted cells, while sarcomatoid cell areas were consistently PAX2-negative (Figure 6A and 6B). Similar PAX2 staining results were obtained from CD44^bright^/CD24^dim^ sorted cell xenografts (Figure 6C and 6D).

### Expression of surface CD105 on the two sorted RCC52 subsets

**Figure 7:**
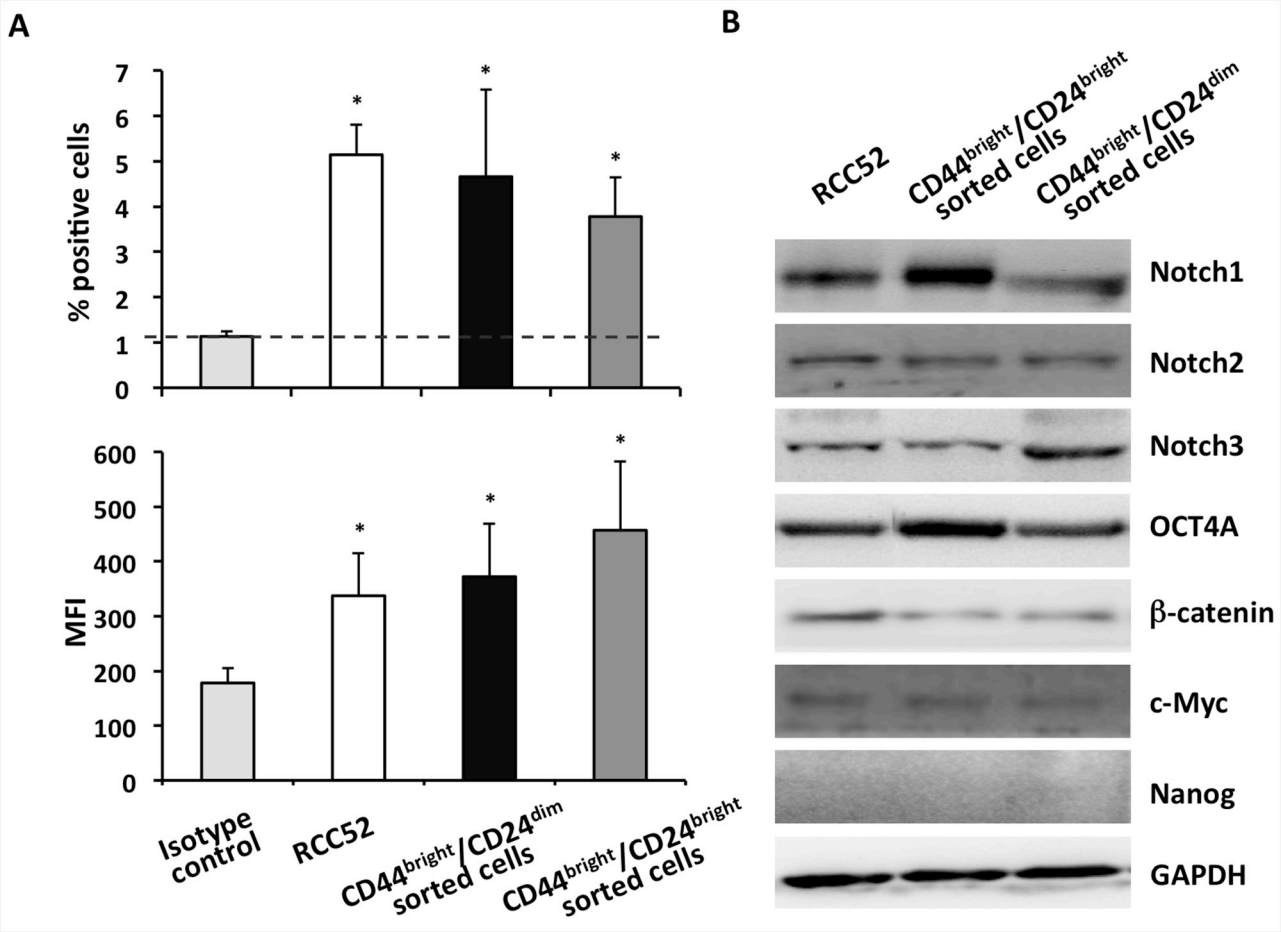
Expression of CD105 and the stemness signatures in sorted RCC52 subsets **A**. Cytofluorometric analysis of surface CD105 expression on sorted RCC52 subsets was performed. Experiments were repeated three times. Results were expressed as % positive cells (upper frame) and mean fluorescence intensity (MFI, lower frame) in a bar representation, where mean ± SD of the 3 experimental results were illustrated. *p < 0.01. **B**. Western blot analysis was conducted to determine the expression of selected stemness genes in the two sorted RCC52 subsets. GAPDH was used as a loading control.

Since the two sorted RCC52 subsets were relatively tumorigenic in NOD/SCID mice with the CD44^bright^/CD24^bright^ fibroblastoid subset yielding a greater tumorogenicity, as demonstrated above, we hypothesized that the RCC CSC marker CD105 should be expressed by both subsets. We conducted cytofluorometric analysis with anti-CD105 mAb, which revealed comparable percentages of surface CD105 positive cells (CD44^bright^/CD24^dim^ sorted cells *vs*. CD44^bright^/CD24^bright^ sorted cells, 4.66 ± 1.9% *vs*. 3.78 ± 0.1%) and comparable MFI levels (372.3 ± 97.1 *vs*. 475.1 ± 126.1) exhibited by the CD44/CD24 sorted subsets (Figure 7A). These results confirmed the existence of CSCs independently in both RCC52 subsets. When the isotype control was tested in the place of anti-CD105 mAb, low percentages of positive cells (~1%) were obtained.

### Expression of stemness gene signatures in the two sorted RCC52 subsets

To add support to the existence of CSCs in both RCC52 subsets, we examined the expression of a panel of selected stemness genes in the two sorted subsets by Western blot. Both subsets expressed significant levels of Notch1, Notch2, Notch3, c-Myc, β-catenin and Oct4A at the protein level, as for the parental line (Figure 7B), but Nanog was not expressed. These results confirmed that each of the two subsets contained its own CSCs.

## DISCUSSION

In our previous study on RCC52 cells with the use of clonal sublines, we obtained two unusual findings [29]. Firstly, in the xenotransplantation study, while RCC52 parental cells gave rise to high tumorigenicity, the epithelioid sublines resulted in low but definitive tumorigenicity and at the same time the fibroblastoid sublines failed to show any detectable tumorigenicity. The extent of tumorigenicity induced by the epthelioid sublines was far less than that by the parental RCC52 cells. Secondly, the tumorigenicity and *in vitro* migratory/invasive potential could be dissociated and independently ascribed to the two subsets based on clonal studies, different from other reported solid tumor types such as squamous cell carcinoma [30], prostate cancer [31], and gastrointestinal carcinoma [13]. In a practical sense, we may have only selected and used a limited number of clonal sublines in that study [29]. These clonal sublines might not represent the whole spectrum of each given subset population of RCC52. We therefore speculated that the conclusions drawn in the study with clonal sublines [29] could be erroneous. With these considerations in mind, we have succeeded in the current study in identifying two mAbs, anti-CD44 and anti-CD24, for the isolation and re-analysis of the two subsets at the whole population level using experimental approaches conducted similar to those used in our previous RCC52 clonal studies [29].

In the plots of this two color cytoflourometric analysis, only the sarcomatoid cell line, RCC52, showed two discrete populations with the phenotypes of CD44^bright^/CD24^bright^ and CD44^bright^/CD24^dim^ corresponding well to respective morphologically fibroblastoid and epithelioid subsets (Figure 1). The remaining five RCC cell lines exhibited only one population with a phenotype of CD44^bright^/CD24^bright^, although the percentages of positive cells and MFI levels were not exactly the same for all these cell lines. The two morphologically distinct subsets could be consistently demarcated at a ratio of approximately 6:1, with the sorted epithelioid subset being the majority, when different *in vitro* passage levels of RCC52 cells were maintained and tested (Figure 2A).

We next determined whether the four epithelioid (E1~E4) and the four fibroblastoid (M1~M4) clonal sublines established in the previous study [29] would fall into one of the two specific phenotypes, i.e. the former to be CD44^bright^/CD24^dim^ and the latter to be CD44^bright^/CD24^bright^. The results of our current study turned out exactly as we expected (Figure 2B), with one exception. This was the epithelioid subline E4, which exhibited one elongated mass covering the two populations, making them in continuum. Such a phenotype of the two populations of E4 subline in fusion was reproducible, as repeated experiments with early and later passaged E4 cells showed similar results (data not shown). This fusion phenotype shown by E4 was not due to the contamination of two morphologically different clones, since we obtained similar results after double and triple cloning procedures were performed. In addition, a sharp DNA content in flow cytometric analysis (not shown), exhibiting the correct cellular morphology and the presence of specific genetic markers, including mutation sites and heterozygosity status at chromosome 15q21 short tandem repeats [28], all point to the genuine clonality of E4. Whether this profile of the image in E4 (Figure 2) represents an incomplete epithelial-to-mesenchymal transition (EMT) is not clear at this time. Our preliminary results revealed that the impact of stress like fetal bovine serum (FBS) deprivation for a period as short as 24 h could lead one sorted subset to convert into another with a greater rate of changes or conversion found from the CD44^bright^/CD24^bright^ phenotype to the CD44^bright^/CD24^dim^ rather than the other way around. Of note, the addition of hypoxic stress over and above FBS deprivation clearly resulted in added effects of the subpopulation conversion with either subset, showing an emergence of two fused phenotypes, like the E4 clone demonstrated in the two color cytofluorometric analysis (Figure 2). These results of mutual convertibility suggest that the two subsets share a common cell origin and are reciprocally convertible. Additional work is required for validation.

The subline E4 has another noteworthy feature. E4 was the only one out of the four epithelioid sublines tested that expressed one of the apoptosis inhibitors, BCL2, which was persistently expressed by all four fibroblastoid, but not by other epithelioid, sublines tested [29]. Based on all these results, E4 may represent a rare variant of RCC52 endowed with a high threshold of resisting a variety of stresses. Although the exact biological significance of this finding is not fully clear, we will continue to investigate this issue as additional data are accumulated.

Apart from the aforementioned discussion on xenotransplantation and migratory/invasive potential, comparative results between the epithelioid and fibroblastoid subsets with respect to additional test parameters (such as phenotyping, *in vitro* growth characteristics, anchorage-independent colony forming ability, and expression of CD105, one of the known RCC stem cell markers [21]) are summarized in Table 2. Notably, surface E-cadherin was the only consistent marker on the epithelioid subset that could be used to distinguish it from the fibroblastoid subset [29]. When the sorted and clonal cells were compared, the trends of results in these parameters generated between the two subsets were similar except for the expression of CD105, secretion of TGF-β1, *in vitro* colony forming ability, and tumorigenicity in NOD/SCID mice (Table 2). The fibroblastoid sublines showed reduced anchorage-independent colony forming ability, TGF-β1 secretion and clear cell convertibility in xenografts compared to their sorted counterparts, likely because the clonal sublines that exhibited these features were not selected and/or were not cloned *in vitro* by the procedures and conditions we used. Therefore, the results obtained with the use of sorted cells are more objective.

**Table 2:** Comparative results of immunophenotypical and biological properties between the RCC52 epithelioid and fibroblastoid subsets using clonally derived sublines *vs*. cytofluorometrically sorted cells

Parameter	Clonal cells		Flow sorted cells	
	Epithelioid	Fibroblastoid	CD44^bright^/CD24^dim^	CD44^bright^/CD24^bright^
**Phenotype**	CD44^bright^/CD24^dim^	CD44^bright^/CD24^bright^	CD44^bright^/CD24^dim^	CD44^bright^/CD24^bright^
**Cell morphology**	Epithelioid	Fibroblastoid	Epithelioid	Fibroblastoid
***In vitro*** **growth pattern**	fast	slow	fast	slow
**Doubling time (h)**	32.0 ± 4.3	50.1 ± 2.8	24.5 ± 0.3	38.4 ± 0.4
**Saturation density (x10^4^/cm^2^)**	5.5 ± 0.6	3.5 ± 0.7	7.0 ± 0.1	7.3 ± 0.3
**Days required for reaching saturation (D)**	2.84 ± 0.07	4.73 ± 0.51	2.89 ± 0.15	3.94 ± 0.08
**Surface HLA class I**	-	-	-	-
**Surface E-cadherin**	21.5 ~ 38.6%	-	18.4%	-
**Surface CD105**	2.79%	1.09%	5.32%	3.89%
**Colony forming ability**	++	-	++++	++
**Migration/Invasion ability**	+	+++	+	+++
**TGF-β1 (10^5^ cells)**	47 ~ 143	17.6 ~ 53.2	154.7 ± 12.3	177.0 ± 31.2
**Xenotransplantation in NOD/SCID mice**	++	-	++++^a^	++++++^a^
**Ability of clear cell conversion^b^**	++	-	+++	++

^a^The CD44^bright^/CD24^bright^ sorted subset grew faster with shorter latent periods than the CD44^bright^/CD24^dim^ sorted subset (see Figure 5A).

^b^Clear cell conversion in areas found in xenografts by immunohistochemtry.

Similar to the results obtained with the use of clonal cells, our *in vitro* observation with sorted cells indicated that the CD44^bright^/CD24^bright^ fibroblastoid subset clearly had a greater migratory/invasive potential than the CD44^bright^/CD24^dim^ epithelioid subset; however, whether differential metastatic activity can be demonstrated *in vivo* remains unknown. This will be tested in xenotransplantation experiments in NOD/SCID mice.

Bussolati *et al*. found a population of CD133^+^ cells in normal renal tissue, which was with the stem cell ability that could differentiate *in vitro* and *in vivo* into endothelial and epithelia cells [19]. Subsequently, they found that the CD133^+^ renal tumor-derived progenitor cells (fibroblastic cells) were not tumorigenic *in vivo*, which is different from the known characteristics of CSC [20]. Of note, none of our epithelioid, fibroblastoid and the parental RCC52 cells was CD133 positive (data not shown). Bussolati *et al*. also found that a subpopulation of CD105^+^ cells in RCC was enriched in CSCs with stem cell characteristics [21]. They isolated a subpopulation of cells expressing the surface marker CD105, representing less than 10% of the tumor mass, and this population could induce tumors in SCID mice. This was what we initially found: CD105 positive for only the RCC52 epithelioid sublines in results obtained with clonal studies [29]. In the present study, with the use of sorted cells, both subsets express their own CSCs in 4~5% of each subset cell population, suggesting the existence of CSCs. This was further confirmed by the expression of some of the embryonic stemness gene markers in each of the subsets as well as in the parental RCC52 cells (Figure 7B).

It has been shown that TGF-β1 can promote tumor progression through effects on stromal fibroblasts [32], endothelial cells [33], and immune cells [34]. TGF-β1 is also known to exhibit both tumor-promoting and suppressing effects, depending on the degree of tumor progression [32]. In breast cancer, TGF-β1 signaling is inhibitory for low-grade tumors, but stimulatory for high-grade tumors and metastasis [33]. In RCC, TGF-β1 expression directly correlates with tumor stage and grade, suggesting its importance in tumor progression [34]. Moreover, studies of Bostrom *et al*. suggest that from histological and immunohistochemical perspectives, sarcomatoid conversion from clear RCC may represent a completed EMT, and TGF-β1 could be an important driving force during the sarcomatoid trans-differentiation of clear cell RCC [9]. The results of the current study clearly showed that being tumorigenic, both subsets were able to secret TGF-β1 *in vitro* in a time-dependent fashion and interestingly at comparable levels (Figure 4C). Nevertheless, the tumor microenvironment in the *in vivo* situation is important; apart from the tumor subsets, other cell types such as stromal fibroblasts, endothelial cells and various immune cells could also play roles in influencing each other.

In view of our own earlier data [28], the divergence of these two subsets might have started at or around the time of sarcomatoid transformation from clear cell RCC in the patient in the case of RCC52. Importantly, cases of clonal divergences and genetic heterogeneity have been reported in clear cell RCC with sarcomatoid transformation [35, 36]. Thus, genetic divergence following neoplastic transformation and progression is reflected in molecular heterogeneity and seen in genetic and retention of heterozygosity (ROH) analysis in clonal evolution of clear cell RCC with sarcomatoid transformation.

Although we could not completely rule out that the phenomenon of EMT or MET might have also occurred between the subsets in RCC52 cells, we did not detect any tumor spheroids or spheres in our *in vitro* studies, as detected in other types of human malignancies, such as breast and colorectal cancers in which the CD44^bright^/CD24^-/dim^ rather than CD44^bright^/CD24^bright^ subset are the markers for CSCs or the niche of the CSCs [13, 16]. With xenotransplantation assays, in this study we clearly demonstrated that both the CD44^bright^/CD24^dim^ sorted and the CD44^bright^/CD24^bright^ sorted subsets exhibited their own CSCs independently. This is a major finding in our current study. Also important, xenografts resulting from the injection of either the CD44^bright^/CD24^dim^ sorted or the CD44^bright^/CD24^bright^ sorted subset could give rise to clear cell morphology. More abundant clear cells could be frequently found at the periphery as well as in the central area of xenografts in a random fashion for the former, as compared with a thin layer of clear cell morphology at a less frequent rate detected in those areas for the latter. Interestingly, the frequency and site of the appearance of PAX2-positive clear cells in the xenografts resulting from parental RCC52 cells injection falls in between those of the two subsets. The clear cell morphology was confirmed to be clear cell RCC as evidenced by their positive PAX2 staining. These results are different from those obtained with clonal sublines, since only parental RCC52 cells as well as the epithelioid, but not fibroblastoid clonal sublines were able to develop xenografts at the injection sites [29]. These xenotransplantation results of clear cell convertibility also point to high plasticity of these two subsets of the RCC52 sRCC cell line. Collectively, our current results suggest strongly that the two sorted subsets of RCC52 have their own CSCs and the ability to exhibit different levels of specific patterns of *MMP* mRNAs expression and their inhibitors (i.e., higher levels of *MMP -2, -9* and *TIMP-2* by the CD44^bright^/CD24^dim^ sorted cells *vs*. higher levels of *MMP-7, -8*, and *TIMP-1* by the CD44^bright^/CD24^bright^ sorted cells), thereby ensuing divergent functional roles in tumor progression.

The xenograft and parental RCC52 original patient tumor sections [28, 29] shared some similarity in terms of the area of clear cells in the majority of sarcomatoid cells, i.e. the areas of clear cells were always stained positively with anti-PAX2 antibody. However, the appearance of clear cell areas in the original patient tumor and that of xenografts resulting from the sorted subsets were clearly different. In the patient's tumor, the clear cell areas appeared to be pushed and squeezed by the apparently more aggressive sarcomatoid cells and clear cells themselves a sign of residual cells.

Increasing evidence indicates that at least some cancer types such as leukemia, breast cancer, colorectal cancer, and hepatocellular carcinoma, are hierarchically organized and follow a CSC model [14, 16, 37]. However, they have rarely been reported regarding CSCs in RCC [18, 19, 21], let alone sRCC. In conclusion, we present the first evidence for the co-existence of CD44^bright^/CD24^dim^ sorted and CD44^bright^/CD24^bright^ sorted subsets, each having its own CSCs in the RCC52 cell line. This requires further validation with additional clear cell-RCC and sRCC clinical specimens and cell lines derived thereof in order to determine the universality for all sRCCs. Recent studies in clear RCCs with sarcomatoid changes have demonstrated anomalous genetic complexity, underlying intratumoral heterogeneity [35, 36]. Selection pressures in the tumor-bearing host either in a natural course of disease or as a result of cancer treatment could act on multiple co-existing subpopulations, perhaps generated by their distinct CSC populations. The process of clonal evolution and tumor progression in patients with cancer suggests that combination approaches targeting all distinct classes of CSCs are needed for successful treatment of sRCC. We hope that the discussion from our current findings will stimulate further studies in this important area of sarcomatoid renal cell carcinoma research.

## MATERIALS AND METHODS

### Cell lines and culture conditions

The human cell lines (HH050, HH244, HH332, HOKN-9, RCC52 and RCC98) were derived from surgically removed primary RCC lesions, except RCC52, which was derived from a regional lymph node. Their origins, sources and characteristics were previously described [28, 29]. All the cell lines were maintained in RPMI-1640 medium (Gibco, Grand Island, NY), containing 2 mM L-glutamine, 100 units/ml penicillin, 100 μg/ml streptomycin, 1 mM sodium private and 10% FBS which had been previously heat-inactivated at 56°C for 30 min. For the clonal sublines, we used the same panel of clonal sublines described previously [28, 29].

### Growth curves

Cells were seeded at 1×10^5^ cells/well in a 6-well plate (diameter 3-cm, Nunc, Roskilde, Danmark) with 2 ml RPMI-1640 medium supplied with 10% FBS at day 0. Then cells were detached from the bottom of wells by light trypsinization at 24 h intervals for 5 days without change of medium, and by the trypan blue exclusion assay cells harvested at each time point were counted under a light microscope. Triplicate wells were used for each time point. The population doubling time and saturation density of cells were calculated from the growth curve. Supernatant of each well was collected and stored at -80°C for the detection of cytokines by enzyme-linked immunesorbent assay (ELISA).

### Cytofluorometric analysis and fluorescence-activated cell sorting

Various cellular components were examined for surface and cytoplasmic expression by immunofluorescence/flow cytometry using a panel of specific monoclonal antibodies (mAbs). A panel of mAbs used for immunophenotyping included BH8.23 (S-K. Liao), CD105 (clone SN6h, Lifespan Bioscuences, Seattle, WA), E-cadherin (clone 67A4, Biodesign, Kennebunk, ME), EMA (clone E29, Dako, Glostrup, Denmark), EpCAM (clone 323/A3, Thermo Fisher scientific, Fremont, CA), HLA class I (clone W6/32, Thermo Fisher scientific), Hsp70 (clone C92F3A-5, Stressgen, Ann Arbor, MI), Lewis Y (Le^y^, clone SDZ-ABL364; from Dr. H. Loibner, SANDOZ Research Institute, Vienna, Austria), N-cadherin (clone 3B9, Zymed, South San Francisco, CA, USA), S100-A4 (polyclonal, Abcam, Cambridge, MA) and vimentin (clone V9, Dako). These reagents were all aliquoted in small volumes and stored at -40°C. The aliquoted tubes containing mAbs were removed from the freezer, diluted to the working concentration 5 μg/ml or a concentration suggested by the manufacturer before use in cytofluorometric analysis, which was performed as previously described [28]. For multiple-color phenotyping, fluorescein isothioyanate (FITC)-conjugated mouse anti-human CD44 (clone G44-26, BD Pharmingen, San Diego, CA) and allophycocyanin (APC)-conjugated mouse anti-human CD24 (clone ML5, Biolegend, San Diego, CA) mAbs were also used according to the manufacturer's instructions. Samples were tested using BD FACSVerse™ (BD Biosciences, San Jose, CA), and data were analyzed by Flowjo software (Tree Star, Ashland, OR). Results are expressed as % positive cells and the relative mean fluorescence intensity (MFI).

For fluorescence-activated cell sorting (FACS), 1×10^7^ cells were collected and then stained with FITC-conjugated mouse anti-human CD44 and APC-conjugated mouse anti-human CD24 mAbs according to the manufacturer's instructions. The cells were washed twice with PBS containing 1~2% FBS and suspended in 1 ml wash buffer. These cells were sorted by BD FACSAria cell sorter (BD Biosciences, Heidelberg, Germany). The sorted cells were maintained in complete medium.

### Cell migration and invasion assays

The cell invasion assay was performed by using a BioCoat Matrigel (BD Biosciences, Bedford, MA) and Transwell^®^ Permeable Support 8 μm PolyCarbonate Membrane in a 24-well plate (Corning Costar, Coring, NY). The Matrigel was diluted with the serum free RPMI-1640 medium to the concentration of 5 mg/ml and added to the center to each cell well inserts with total volume 100 μl for 1 h at 37°C. Cells (1×10^5^) were suspended in the RPMI-1640 medium containing 1% FBS and seeded into the upper chamber. The lower chamber contained complete culture medium supplied with 10% FBS. The cell invasion ability was determined by counting cells in the lower chamber that had previously passed through the Matrigel-coated membrane. The cell migration assay was performed to add the cells directly without the coating process of the Matrigel. The cells at the bottom of the membrane were fixed in methanol and stained by Giemsa stain (Sigma-Aldrich, St. Louis, MO). Cells in three randomly selected microscopic fields (× 400) were counted. Each experiment was repeated three times.

### ELISA

The quantification of TGF-β1 in the spent media was performed using ELISA kits (R&D systems, Minneapolis, MN, USA) according to the manufacturer's instructions. The spent media of cultures were harvested at day 1, 3 and 5 after initiation of culture as in growth curve studies.

### Anchorage-independent colony-forming assay

For anchorage-independent colony forming assays, 6-well plates (Corning Costar) were previously coated with 1% ultralow agarose (Sigma-Aldrich) in normal supplemented culture medium to avoid cells seeded to the bottom. Each cell populations were suspended at concentration of 5×10^3^ cells/ml in normal supplemented culture medium with 0.5% ultralow agarose and then plated on top of coated 1% ultralow agarose basal layer (N = 3 for each cell subset). One ml normal supplemented culture medium was added on top of the concreted cell layer to avoid loss of water in cell layer by evaporation. After 20 days culture at 37°C with a humidified atmosphere containing 5% CO_2_ and 95% air, the colonies developed in agar were stained with crystal violet overnight, photographed, and then manually counted in the entire field of each well.

### qPCR

Total RNA was extracted separately from cells using TRIzol reagent (Invitrogen, Carlsbad, CA) according to the manufacturer's instructions. Total RNA was eluted in RNase free water and stored at -80°C. Total RNA was reversely transcribed to cDNA using a ReverTra Ace Set (Toyobo, Osaka, Japan) according to the manufacturer's instructions and stored at -20°C. The *matrix metalloproteinases* (*MMPs*) genes and their primers were listed in Table 3. The reactions were carried out by 5x EvaGreen qPCR Mix with ROX (TOOLS Biotechnology Co., Ltd. Taiwan) according to manufacturer's instructions. After initial denaturation at 95°C for 15 min, 40 cycles of denaturation at 95°C for 15 sec, annealing at different temperatures from 52 to 58°C for 20 sec and extension at 72°C for 20 sec were performed. qPCR reactions were done in triplicate. The relative mRNA expression levels were calculated using comparative Ct (ΔΔCt) method, with β-actin as a reference gene.

**Table 3:** Specific primers for MMPs and β-actin genes

Primer	Sequence
*MMP-1*	Forward:5’-ATGCTGAAACCCTGAAGGTG-3’Reverse: 5’-GAGCATCCCCTCCAATACCT-3’
*MMP-2*	Forward: 5’-TTTCCATTCCGCTTCCAGGGCAC-3’Reverse:5’-TCGCACACCACATCTTTCCGTCACT-3’
*MMP-7*	Forward: 5’-TCCAACCTATGGAAATGGAGA-3’Reverse: 5’-GGAGTGGAGGAACAGTGCTT-3’
*MMP-8*	Forward: 5’-CCATCTATGGACTTTCAAGCAAC-3’Reverse: 5’-TTGGAAGGGATGGCCAGAATAG-3’
*MMP-9*	Forward: 5’-TTGACAGCGACAAGAAGTGG-3’Reverse: 5’-GCCATTCACGTCGTCCTTAT-3’
*TIMP-1*	Forward: 5’-CAGACCACCTTATACCAGCGT-3’Reverse: 5’-GATAAACAGGGAAACACTGTGC-3’
*TIMP-2*	Forward: 5’-TATCTACACGGCCCCCTCCT-3’Reverse: 5’-ACCCAGTCCATCCAGAGGCA-3’
*β-actin*	Forward: 5’-GAGCGCGGCTACAGCTT-3’Reverse: 5’-TCCTTAATGTCACGCACGATTT-3’

### Xenotransplantation in immunodeficient mice

Tumorigenicity assay was carried out in NOD/SCID immunodeficiency mice. Six-week-old female NOD/SCID mice (N = 4 or N = 5) were purchased from the BioLASCO, Taiwan Co., Ltd. Tumor cells were collected by light trypsinization, followed by washing once in PBS. Cells (1×10^6^ and 5×10^6^ cells/0.1 ml PBS) were injected subcutaneously at a site above the hind leg of each mouse. The animals were examined every 2 days for a period of 132 days to monitor the growth of tumors. The volume of palpable tumor nodules was calculated according to the formula: volume (mm^3^) = 0.4x*a*x*b*^2^, where *(a)* is the major diameter and *(b)* is the minor diameter perpendicular to the major one. At the end of observation, animals were sacrificed and tumors were removed for either setting up cell culture and being fixed in formaldehyde for immunohistochemistry. All animal experiments were conducted in accordance with the Guide for the Care and Use of Laboratory Animals of the National Institute of Health and following the Institutional Animal Care and Use Committee (IACUC) protocol authorized by Chang Gung University, Taiwan.

### Histology and immunohistochemistry

Sections (5-μm in thickness) of formalin-fixed, paraffin-embedded tumor blocks obtained from the xenograft were processed. Hematoxylin-Eosin (H&E, Sigma-Aldrich) staining was performed to detect histology details of the tissue section. Prior to immunostaining, the deparaffinized slides were subjected to an antigen retrieval process by dipping the slides in a beaker containing 0.01 M sodium citrate (pH 6.0) in a boiling state on a hotplate. Following a 20 min incubation, the beaker was removed from the hotplate and allowed to cool down to room temperature for 20 min. Slides were washed once in PBS and stained using the UltraVision Quanto Detection System HRP DAB kit (Lab Vision Corporation, Fremont, CA), according to the manufacturer's instructions. Anti-PAX2 polyclonal Ab (Invitrogen) was used in this study.

### Western blotting

Cell lines were lysed in PRO-PREPTM Protein Extraction Solution (iNtRON Biotechnology, Sangdaewon-Dong, KOREA) according to the manufacturer's instructions. Protein concentration was determined by Qubit^®^protein assay kit (Invitrogen). Total cell lysates (30 μg) were separated electrophoretically by an 8% or 10% polyacrylamide SDS-PAGE gel and transferred to polyvinylidene fluoride (PVDF) membrane (Immobilon P, Millipore, Bedford, MA, USA). Membranes were blocked with 5% non-fat milk in TBS-T buffer (150 mM NaCl, 10 mM Tris/pH 8.0, and 0.05% Tween 20) at room temperature for 1 h. The membranes were then immunoblotted with primary antibody overnight at 4°C, followed by incubation with secondary antibodies for 1 h at room temperature. Blots were visualized by a chemiluminescence ECL system (Millipore Corporation, USA). The antibodies used for immunoblots included Notch1 (clone D6F11, Cell Signaling Technology, Beverly, MA), Notch2 (clone D67C8, Cell Signaling Technology), Notch3 (clone D11B8, Cell Signaling Technology), OCT4A (clone 1C4B6, Proteintech, Chicago, IL), β-catenin (cloneE7, EMD Millipore, Solna Sweden), c-Myc (clone 9E10, Santa Cruz Biotechnology, Santa Cruz, CA), Nanog (clone D73G4, Cell Signaling Technology) and GAPDH (clone 6C5, EMD Millipore). Of note that floating (F) cells of the UP-LN1 cell line expressed the stemness genes indicated above in our previous study [38]. Thus we used that F cells as positive control cells in the current Western blot experiments (data not shown).

### Statistical analysis

All experiments were performed in triplicates or greater. Data are presented as the mean ± SD of the indicated number of independent experiments. Student's *t*-test was employed to evaluate the results. Differences were considered statistically significant with *p-*values of less than 0.05.
